# Adenoma Detection and the Endoscopist: Assessing the Effects of Insourcing in UK Endoscopy Services

**DOI:** 10.7759/cureus.85272

**Published:** 2025-06-03

**Authors:** Mo Thoufeeq, Cyrias Makkattil, Nadha Rahim, Bilal Azam, Nilanga Nishad

**Affiliations:** 1 Gastroenterology, Sheffield Teaching Hospitals NHS Foundation Trust, Sheffield, GBR; 2 Internal Medicine, Sheffield Teaching Hospitals NHS Foundation Trust, Sheffield, GBR; 3 Gastroenterology and Hepatology, Sheffield Teaching Hospitals NHS Foundation Trust, Sheffield, GBR

**Keywords:** adenoma detection, colonoscopy, colorectal cancer, endoscopist role, insourcing, polyp detection rate

## Abstract

Introduction

Endoscopy services in the UK often supplement staffing through insourcing, yet the impact of this model on diagnostic outcomes remains unclear. This study evaluates differences in polyp and colorectal cancer detection between standard hospital endoscopists and insourced endoscopists at Sheffield Teaching Hospitals NHS Foundation Trust.

Methods

A retrospective observational study was conducted from March 1, 2022, to February 28, 2024, involving 2,173 diagnostic colonoscopies. All insourced procedures (n=1,205) were included, while a systematic one-in-five sampling approach yielded 968 hospital-based procedures. Only diagnostic colonoscopies were considered, excluding therapeutic, bowel cancer screening programme (BCSP), and inflammatory bowel disease (IBD) surveillance cases. All polyps identified during procedures were histologically matched through pathology records. Demographics and histological findings were compared between the two groups using chi-squared and t-tests.

Results

Patient demographics were similar across groups. Hospital endoscopists had significantly higher detection rates for tubular adenomas (21.2% vs. 14.9%, p<0.001), hyperplastic polyps (8.5% vs. 5%, p=0.001), and colorectal carcinoma (1.7% vs. 0.5%, p=0.003). Insourced endoscopists detected more villous/tubulovillous adenomas (3.2% vs. 1.3%, p=0.004). A higher percentage of procedures by insourced endoscopists yielded no polyp findings (73% vs. 63.5%, p<0.001).

Conclusion

Hospital endoscopists demonstrated higher detection rates for most clinically significant polyps and colorectal cancer. The findings suggest potential variability in diagnostic yield based on the endoscopist's role, warranting further evaluation of training, audit, and performance standards across staffing models.

## Introduction

The United Kingdom is under enormous pressure as it is nearly failing to cater to the service demands for endoscopies [1-3]. Regional variabilities in endoscopy services are apparent, with some areas performing significantly better than others [4].

To increase capacity, many services have adopted special initiatives, such as extending weekday and weekend hours with overtime pay, subcontracting to suppliers who employ locum or agency staff to use NHS facilities for procedures (insourced), and referring patients to external providers (outsourcing) [5]. In 2021, 46% of UK endoscopy services used insourced, and 53% conducted weekend lists [2]. Approximately 25% of units engaged in both "insourced" (bringing in external staff to perform procedures within their facilities) and "outsourcing" (referring patients to external services for procedures) [4]. The 2019 Joint Advisory Group on Gastrointestinal Endoscopy (JAG) UK survey showed that 17.2% of services were outsourcing activity to external providers, while 36.1% reported insourced activity [6]. While these measures have helped to alleviate some of the pressure, they also raise important questions about the long-term sustainability of endoscopy services in the UK.

Quality indicators, such as the polyp detection rate (PDR), are used to assess and improve the performance of colonoscopies. The PDR is influenced by various factors, including the quality of bowel preparation, the caecal intubation rate, and withdrawal time [7]. In specific cases, such as inflammatory bowel disease (IBD), optimal colonoscopy technique is critical for detecting dysplasia [8]. The adenoma detection rate (ADR) is a key quality measure for colonoscopy, but it has limitations. It only accounts for whether an endoscopist finds at least one adenoma per patient, not their ability to detect multiple adenomas. This can lead to the "one and done" phenomenon, where endoscopists might reduce their examination quality after finding a single adenoma [9]. The ADR and mean number of polyps (MNP) per procedure were lower on Saturdays and evenings than on weekdays [10].

A recently published UK national endoscopy database study, conducted in 2019, on the MNP detected and PDR found that weekend-insourced services had a lower MNP and PDR. They concluded that this might affect the quality of weekend services, which needs further investigation into the cause [11,12]. Another study by Thompson et al., comparing weekend services with weekday services, found no statistical difference in outcomes, such as PDR and caecal intubation rates [13]. This study was planned to further evaluate the outcomes of weekend-insourced colonoscopies, comparing the roles of endoscopists with other factors.

## Materials and methods

This retrospective observational study aimed to compare polyp and colorectal cancer detection outcomes based on the role of the endoscopist, standard hospital-employed versus insourced, within Sheffield Teaching Hospitals NHS Foundation Trust, over a two-year period from March 1, 2022, to February 28, 2024. The primary objective was to evaluate the impact of the endoscopist type on the histologically confirmed detection of polyps and colorectal carcinomas.

During this period, a total of 20,591 colonoscopies were performed at the Trust. Of these, 13,498 diagnostic colonoscopies met the inclusion criteria. Excluded procedures included therapeutic colonoscopies at booking, bowel cancer screening programme (BCSP) colonoscopies, and IBD surveillance procedures involving dye spray.

For the purposes of this study, a total of 2,173 colonoscopy procedures were analyzed. All 1,205 insourced colonoscopies performed during the study period were included. A systematic sampling approach was applied to the hospital-based endoscopies: every fifth diagnostic colonoscopy performed by standard (in-hospital) endoscopists, beginning from the earliest procedure in the study period, was selected until a sample of 968 was reached. This ensured chronological coverage and reduced selection bias.

Endoscopists were classified into two main categories based on their roles: (1) standard (hospital) endoscopists - endoscopists regularly employed and practising at Sheffield Teaching Hospitals, including consultants, clinical fellows, and clinical endoscopists; and (2) insourced endoscopists - visiting endoscopists not primarily employed by the Trust, contracted on a temporary basis from other NHS or private organizations to increase service capacity.

Indications for colonoscopy included a wide range of diagnostic prompts such as altered bowel habits (constipation, diarrhoea, or alternating symptoms), rectal bleeding, abdominal pain, unexplained weight loss, iron deficiency anaemia or low ferritin, elevated faecal calprotectin, family history of colorectal cancer or polyps, positive faecal immunochemical testing (FIT), follow-up of previous colorectal neoplasia, or abnormal imaging findings.

This study focused solely on patient demographic characteristics and the histological outcomes of polyps removed during the procedures. For each colonoscopy included, all polyps identified were matched with their corresponding histopathological diagnosis, confirmed through the hospital's pathology database.

While initial protocol design included procedural quality markers such as caecal intubation rates, scope withdrawal time, and bowel preparation scoring (e.g., Boston Bowel Preparation Score), these were excluded from the final analysis. The study's refined scope centred exclusively on comparing polyp and cancer histology outcomes between the two endoscopist groups.

PDR was defined as the proportion of colonoscopies in which at least one polyp was histologically confirmed. Cancer detection was based on histopathological confirmation of colorectal adenocarcinoma. Comparative statistical analysis was performed using chi-squared tests for categorical variables. Statistical significance was defined as p < 0.05.

Ethical approval for the study was granted under IRAS (Integrated Research Application System) number 326218 at Sheffield Teaching Hospitals NHS Trust. All patient data were fully anonymized to maintain confidentiality and in accordance with the principles outlined in the Declaration of Helsinki.

## Results

Patient characteristics

In both groups, the gender distribution was similar, with females comprising 54.1% of the hospital endoscopist group and 55.3% of the insourcing group. Males accounted for 45.8% and 44.5%, respectively. Age distributions were broadly comparable. The 60-69 age group was most represented in the hospital cohort (21.6%), while both the 60-69 and 70-79 age groups were highest in the insourcing group (each 19.7%). A slightly higher proportion of patients aged 80 years or older underwent colonoscopy in the insourcing group (8.6%) compared to the hospital group (6.2%) (Table 1).

**Table 1:** Patient characteristics for the colonoscopy according to the endoscopist's role (standard vs. agency endoscopist or insourced)

Variables	Hospital Endoscopist		Insourcing Endoscopist	
	Frequency	Percent	Frequency	Percent
Gender				
Female	524	54.1	667	55.3%
Male	443	45.8	538	44.5%
Total	968	100	1205	100%
Age category				
<39 years	196	20.2	230	19.1%
40–49 years	131	13.5	175	14.5%
50–59 years	187	19.3	222	18.4%
60–69 years	209	21.6	237	19.7%
70–79 years	185	19.1	237	19.7%
> 80 years	60	6.2	104	8.6%
Total	968	100	1205	100

Hospital endoscopists had higher detection rates for tubular adenomas (21.2% vs. 14.9%), hyperplastic polyps (8.5% vs. 5%), and colorectal carcinoma (1.7% vs. 0.5%), all with statistically significant differences (p < 0.01). In contrast, insourcing endoscopists detected more villous/tubulovillous adenomas (3.2% vs. 1.3%, p = 0.004). No polyp was found in 73% of insourcing cases compared to 63.5% with hospital endoscopists (p < 0.001). Detection rates for sessile serrated and serrated hyperplastic polyps were low and not significantly different between groups (Table 2).

**Table 2:** Polyp detection rates based on the role of the endoscopist

Polyp Subtype	Hospital Endoscopist		Insourcing Endoscopist		Chi-Square Value	Significance
	Frequency	Percent	Frequency	Percent		
Tubular adenoma	206	21.2	179	14.9%	16.3	<0.001
Villous/tubulovillous adenoma	13	1.3	39	3.2%	8.3	0.004
Serrated hyperplastic	10	1	1	0.1%	N/A	N/A
Sessile serrated lesion	23	2.2	19	1.6%	1.6	0.2
Other polyp	3	0.3	7	0.6%	N/A	N/A
No polyp	615	63.5	880	73%	26.1	<0.001
Colorectal carcinoma	16	1.7	6	0.5%	9	0.003
Hyperplastic polyp	82	8.5	60	5%	12	0.001

## Discussion

This study reveals notable disparities in polyp and colorectal cancer detection based on the role of the endoscopist, whether they are employed in-hospital (standard) or brought in via insourcing arrangements. Hospital-based endoscopists achieved significantly higher detection rates of tubular adenomas, hyperplastic polyps, and colorectal carcinomas compared to their insourced counterparts. These findings suggest that the nature of the endoscopist's engagement and potential differences in training, procedural thoroughness, and familiarity with institutional practices may impact diagnostic outcomes.

The PDR, particularly for tubular adenomas, a surrogate for ADR, was significantly higher among hospital endoscopists (21.2% vs. 14.9%, p < 0.001). This aligns with previous research emphasizing the strong inverse relationship between ADR and interval colorectal cancer incidence and mortality, reinforcing ADR as a key quality metric for colonoscopy performance [14,15]. Studies have consistently shown that an increase in ADR by just 1% is associated with a 3% decrease in the risk of interval colorectal cancer [16].

In contrast, insourced endoscopists had a significantly higher detection rate of villous/tubulovillous adenomas (3.2% vs. 1.3%, p = 0.004), which may reflect a variation in lesion recognition or documentation standards rather than overall procedural quality. However, the higher rate of "no polyp" findings in insourced procedures (73% vs. 63.5%, p < 0.001) raises concerns about the overall sensitivity and thoroughness of examinations performed by this group.

Colorectal carcinoma detection was also significantly lower among insourced endoscopists (0.5% vs. 1.7%, p = 0.003). This result is particularly concerning as it suggests a potential gap in cancer recognition that could impact patient prognosis. Early detection of malignancy during colonoscopy is critical, and missed lesions during lower-quality examinations are associated with higher rates of interval cancers and adverse outcomes [17,18].

Several factors may contribute to the observed differences. Withdrawal time, a well-established quality indicator, has been strongly linked with adenoma detection. Barclay et al. found that colonoscopies with withdrawal times of six minutes or more had significantly higher ADRs [19]. Hospital-based endoscopists, being more integrated within local quality improvement protocols, may be more likely to adhere to recommended withdrawal times and institutional best practices, leading to more effective lesion detection [20]. Nishad et al. (2024) reported that the mean withdrawal time for the hospital endoscopists was 11.6 minutes (SD = 5.8), while the insourced group had a mean time of 9.5 minutes (SD = 3.7) (p < 0.001) [21].

Furthermore, the use of adjunctive techniques, such as Buscopan and sedation, also varies among endoscopist groups and may affect visualization quality and lesion detection. A study by East et al. suggested that optimal bowel relaxation using agents like hyoscine butylbromide can improve mucosal visualization, thereby increasing detection rates for subtle lesions such as sessile serrated adenomas [22]. A study by Nishad et al. in 2024 found that the use of Buscopan was lower among the insourcing endoscopists [21].

Training background, procedural volume, and familiarity with institutional pathways likely play critical roles. Hospital endoscopists are more embedded within a continuous quality assurance environment, which supports better performance through feedback, audits, and peer reviews. Insourced endoscopists, often rotating across sites, may lack this integration, leading to variability in performance [23]. Indeed, Kaminski et al. underscored that endoscopist-level ADR is influenced by both individual skill and institutional support systems [24].

Interestingly, although age distribution was broadly similar between the two groups, with slightly more older patients (>80 years) in the insourcing group (8.6% vs. 6.2%), subgroup analysis indicated that age was not a confounding factor in the lower PDR among insourced endoscopists. This suggests that the quality gap is unlikely to be explained solely by patient demographics.

These findings echo broader concerns about the trade-offs between increasing service capacity through insourcing and maintaining procedural quality. While insourcing addresses backlogs and demand pressures, the reduced detection rates observed in this study may compromise long-term patient outcomes if not addressed through robust governance and quality monitoring.

This study has several limitations that must be acknowledged. First, as a retrospective observational analysis, it is inherently subject to selection bias and confounding factors that may not have been fully accounted for, despite efforts to match patient groups. Second, although procedural metrics such as PDR and ADR were used as proxies for quality, other important factors, such as bowel preparation scores, withdrawal time adherence, and the use of adjunct techniques, were not considered. Third, the study did not stratify results based on individual endoscopist performance or training background, which may have influenced outcomes independently of employment type. Fourth, while age distribution was comparable between groups, other patient-level variables, such as comorbidities, prior colonoscopy history, and genetic risk factors, were not controlled for and may have contributed to the detection variability. Lastly, as the analysis was limited to data from UK services, the findings may not be generalizable to international settings with differing healthcare structures or quality assurance protocols.

This study further helps to solidify the fact raised by a few recent studies that there may be a compromise in the quality of endoscopy while trying to meet service demands. Therefore, we believe the NHS has a new challenge: increasing service provision while maintaining the quality of its services.

## Conclusions

Our results support the growing evidence that colonoscopy quality is significantly influenced by endoscopist role and integration within institutional frameworks. Given the apparent associations between PDR, ADR, and cancer prevention, NHS trusts must weigh the benefits of insourcing against the risks of reduced diagnostic quality and accuracy. Enhancing oversight, standardizing procedural techniques, and integrating insourced staff into continuous quality improvement initiatives could help mitigate these disparities.
